# microRNA-363-3p reduces endothelial cell inflammatory responses in coronary heart disease via inactivation of the *NOX4*-dependent p38 MAPK axis

**DOI:** 10.18632/aging.202721

**Published:** 2021-03-19

**Authors:** Tao Zhou, Suining Li, Liehong Yang, Daokang Xiang

**Affiliations:** 1 Department of Cardiac Surgery, Guizhou Provincial People’s Hospital, Guiyang 550002, P.R. China

**Keywords:** microRNA-363-3p, NADPH oxidase 4, p38 MAPK, coronary heart disease, endothelial cell

## Abstract

Coronary heart disease (CHD) is one of the leading causes of heart-associated deaths worldwide. This study aimed to investigate the mechanism by which microRNA-363-3p (miR-363-3p) regulates endothelial injury induced by inflammatory responses in CHD. The expression patterns of miR-363-3p, NADPH oxidase 4 (NOX4), and p38 MAPK/p-p38 MAPK were examined in an established atherosclerosis (AS) model in C57BL/6 mice and in isolated coronary arterial endothelial cells (CAECs) after gain- or loss-of-function experiments. We also measured the levels of inflammatory factors (IL-6, ICAM-1, IL-10 and IL-1β), hydrogen peroxide (H_2_O_2_), and catalase (CAT) activity, followed by detection of cell viability and apoptosis. In AS, miR-363-3p was downregulated and NOX4 was upregulated, while miR-363-3p was identified as targeting NOX4 and negatively regulating its expression. The AS progression was reduced in NOX4 knockout mice. Furthermore, miR-363-3p resulted in a decreased inflammatory response, oxidative stress, and cell apoptosis in CAECs while augmenting their viability via blockade of the p38 MAPK signaling pathway. Overall, miR-363-3p hampers the NOX4-dependent p38 MAPK axis to attenuate apoptosis, oxidative stress injury, and the inflammatory reaction in CAECs, thus protecting CAECs against CHD. This finding suggests the miR-363-3p-dependent NOX4 p38 MAPK axis as a promising therapeutic target for CHD.

## INTRODUCTION

Coronary heart disease (CHD) is a highly prevalent cardiovascular disease primarily caused by atherosclerosis (AS), due to the passive accumulation of cholesterol and the proliferation of inflammatory macrophages in blood vessel walls [1]. As an active inflammatory disease, AS is characterized by inflammation of the arterial wall, which is mediated by an array of thrombotic factors, growth factors, cytokines, and vasoactive molecules [2]. Endothelial cells are vital for the maintenance of the functional integrity of the vascular wall and nonthrombogenic blood-tissue reactions and serve as modulators of immune responses [3]. Furthermore, endothelial dysfunction is a primary contributing factor to several cardiovascular diseases [4, 5]. Indeed, there is a clear correlation between the atherosclerotic burden and various other risk factors due to the extent of endothelial dysfunction [3]. Due to a persistent lack of clinically approved therapeutic options for ameliorating endothelial cell inflammation, there is an urgent need for the development of novel approaches derived from insights into the underlying pathophysiological mechanisms.

MicroRNAs (miRs) are noncoding nucleotide sequences that modulate gene expression, thus potentially altering endothelial cell phenotypes and vascular smooth muscle function in physiological and disease contexts through translational repression and/or effects on posttranscriptional degradation [6]. miRs can function as direct or indirect modulators at posttranscriptional levels in vascular biology, such that miRs with specific effects on vascular remodeling have emerged as promising targets in treatment [7]. Other reports have also identified vital roles for miRs in the pathogenesis of AS and CHD [8, 9]. Interestingly, extensive miR expression profiling and functional knockdown experiments in human embryonic stem cell-derived cardiomyocytes have identified miR-363 as a possible upstream negative regulator of the left ventricular specification transcription factor HAND1 [10, 11]. miR-363-3p is a 22 bp linear RNA sequence (AATTGCACGGTATCCATCTGTA) (https://www.ncbi.nlm.nih.gov/nuccore/KT921595.1). Moreover, miR-363-3p has been reported to exert neuroprotective effects against ischemic stroke in females [12]. miR-363-3p can influence the angiogenic characteristics of endothelial cells, regulate the expression of angiogenesis-related factors, and control the interaction between hematopoietic cells and endothelial cells [13]. The Targetscan website (http://www.targetscan.org) has predicted that NADPH oxidase 4 (*NOX4*) is a target of miR-363-3p. The *NOX4* gene encodes an oxidoreductase that can facilitate the conversion of molecular oxygen into different reactive oxygen species (ROS), with diverse consequences on the biology of endothelial cells as well as on blood pressure [14]. Overexpression of *NOX4* has been observed in several pulmonary and cardiovascular diseases, including AS, pulmonary hypertension, and pulmonary fibrosis [15, 16]. Moreover, the downregulation of *NOX4* expression induced by dihydrotanshinone I (a natural compound extensively used for treating cardiovascular diseases) may impede atherosclerotic plaque formation and reduce oxidative stress in atherosclerotic mice [17]. Atorvastatin has demonstrated its inhibitory role in the homocysteine-induced oxidative stress and apoptosis in endothelial progenitor cells via the inactivation of the *NOX4*-dependent p38 mitogen-activated protein kinase (MAPK) signaling pathway [18]. An existing study has illustrated the relationship between the pathogenesis of cardiovascular diseases and the p38 MAPK signaling pathway [19], the inhibition of which is considered to be a therapeutic target for vascular remodeling [20]. On the basis of the aforementioned literature, we hypothesized that miR-363-3p may inhibit endothelial cell inflammation-induced injuries by targeting *NOX4* via modulation of the p38 MAPK signaling pathway. To test this hypothesis, we examined the effects of miR-363-3p-mediated inhibition of *NOX4* and the subsequent modulation of the p38 MAPK signaling pathway on the inflammatory injury of endothelial cells following CHD.

## RESULTS

### Increased blood lipid and calcium levels in AS mice are reduced by a *NOX4* knockout

Blood lipid and calcium levels of mice in each group were quantified using an Abbott Aeroset^TM^ biochemical analyzer and are summarized in Table 1. In contrast to the levels observed in the normal mouse group, the serum levels of total cholesterol (TC), triglyceride (TG), low-density lipoprotein cholesterol (LDLC) and Ca^2+^ were increased in the AS group (p < 0.05), whereas there was no significant difference in the serum level of high-density lipoprotein cholesterol (HDLC) (p > 0.05). However, the aforementioned indicators did not differ among the normal (normal mice), N-*NOX4*^-/-^ (*NOX4* knockout mice), and AS-*NOX4*^-/-^ (*NOX4* knockout mice following AS induction) groups (p > 0.05). These results suggested that the elevated blood lipid and calcium levels in AS mice could be prevented by the *NOX4* knockdown.

**Table 1:** Levels of TC, TG, LDLC, HDLC and Ca^2+^ were increased in AS mice and could be reduced by *NOX4* knockout (mmol/L).

**Group**	**TC**	**TG**	**LDLC**	**HDLC**	**Ca^2+^**
Normal	4.41 ± 0.34	1.21 ± 0.39	1.13 ± 0.29	2.26 ± 0.24	2.39 ± 0.38
AS	16.87 ± 1.39^*^	4.22 ± 0.57^*^	5.82 ± 0.99^*^	2.61 ± 0.31	3.57 ± 0.67^*^
N-*NOX4*^-/-^	5.64±0.31	1.53 ± 0.31	1.21 ± 0.11	2.21 ± 0.20	2.41 ± 0.22
AS-*NOX4*^-/-^	7.62 ± 0.27	2.22 ± 0.57	2.71 ± 0.11	2.26 ± 0.21	2.71 ± 0.25

Note: *NOX4*, NADPH oxidase 4; AS, atherosclerosis; TC, total cholesterol; TG, triglyceride; LDLC, low density lipoprotein cholesterol; HDLC, high density lipoprotein cholesterol; the data were analyzed by one-way ANOVA with Tukey's test; n = 10; * *p* < 0.05 *vs.* the normal group.

### miR-363-3p expression is downregulated and *NOX4* mRNA expression is upregulated in AS mice, and *NOX4* knockout decreases the CD34 level in endothelial cells and aortic plaque formation in AS mice

**Figure 1:**
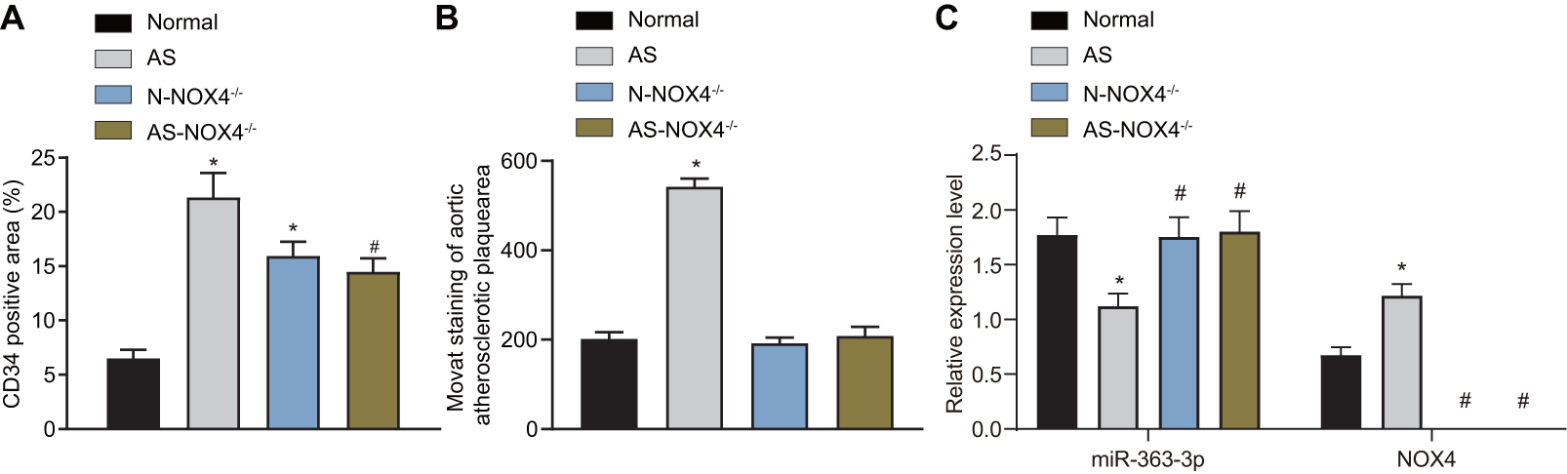
**miR-363-3p and CD34 expressions are downregulated and *NOX4* mRNA expression is upregulated in AS mice and the formation of aortic plaques in AS mice could be prevented by *NOX4* knockout.** (**A**) quantification of positive expression of CD34 protein in mice; (**B**) quantification of the aortic plaque area; (**C**) the expression of miR-363-3p and *NOX4* mRNA as detected by RT-qPCR; The data were analyzed by one-way ANOVA with Tukey's test; n = 10; * p < 0.05 vs. the normal group; # p < 0.05 vs. the AS group; AS, atherosclerosis; *NOX4*, NADPH oxidase 4.

CD34 is the surface marker of mature endothelial cells, and CD34 immunostaining in coronary artery sections can reflect the integrity of the vascular endothelium [21]. Therefore, we undertook immunohistochemical staining to detect the level of the coronary aorta-specific marker, CD34. As depicted in Figure 1A, the level of CD34 was increased in the endothelial cells in the AS and the AS-*NOX4*-/- groups when compared to the normal group but was reduced in the AS-*NOX4*^-/-^ group compared to the AS group (p < 0.05), suggesting that the loss of *NOX4* might attenuate endothelial cell injury caused by AS. Movat’s pentachrome staining was used to stain the nuclei and elastic fibers in black, the proteoglycans in blue, the collagen fibers and the reticular fibers in yellow, the muscles in red, and the fibrin and the red cells in bright red, thereby demonstrating the specific areas of artery trunk lesions in the head and arm of the mice in each group (Figure 1B, Table 2). The plaque area, external elastic membrane area and plaque area/vascular area were increased in the AS group compared with the normal group (p < 0.05). There were no significant differences in the plaque area, external elastic membrane area, or the plaque area/vascular area among the normal, N-*NOX4*^-/-^ and AS-*NOX4*^-/-^ groups (p > 0.05). Overall, these results showed that AS may lead to an increased level of CD34 in endothelial cells and enhance aortic plaque formation in mice, which could be rescued by *NOX4* knockout.miR-363-3p expression and the mRNA expression of *NOX4* in coronary artery tissues in the normal, AS, N-*NOX4*^-/-^ and AS-*NOX4*^-/-^ groups, as detected by reverse transcription quantitative polymerase chain reaction (RT-qPCR), are shown in Figure 1C. miR-363-3p expression was downregulated, while the *NOX4* mRNA expression was upregulated in the coronary artery tissues in the AS group compared to the normal group (p < 0.05). There were no significant differences regarding the miR-363-3p expression in coronary artery tissues among the normal, N-*NOX4*^-/-^ and AS-*NOX4*^-/-^ groups. In addition, *NOX4* mRNA expression was absent in the N-*NOX4*^-/-^ and AS-*NOX4*^-/-^ groups (p > 0.05). The aforementioned results indicated poor miR-363-3p expression and an amplified *NOX4* expression in AS mice.

**Table 2:** The aortic plaque formation in AS mice could be prevented by *NOX4* knockout.

**Group**	**Plaque area (× 10^3^ um^2^)**	**Plaque area/Vascular area**
Normal	201.54 ± 15.27	0.46 ± 0.05
AS	542.43 ± 17.69^*^	0.72 ± 0.08^*^
N-*NOX4*^-/-^	191.76 ± 12.85	0.53 ± 0.04
AS-*NOX4*^-/-^	208.46 ± 20.33	0.49 ± 0.07

Note: The data were analyzed by one-way ANOVA with Tukey's test; n = 10; ^*^ *p* < 0.05 *vs.* the normal group; AS, atherosclerosis; *NOX4*, NADPH oxidase 4.

### *NOX4* knockout decreases phosphorylated (p)-p38 MAPK/p38 MAPK in the coronary artery tissues of AS mice

**Figure 2:**
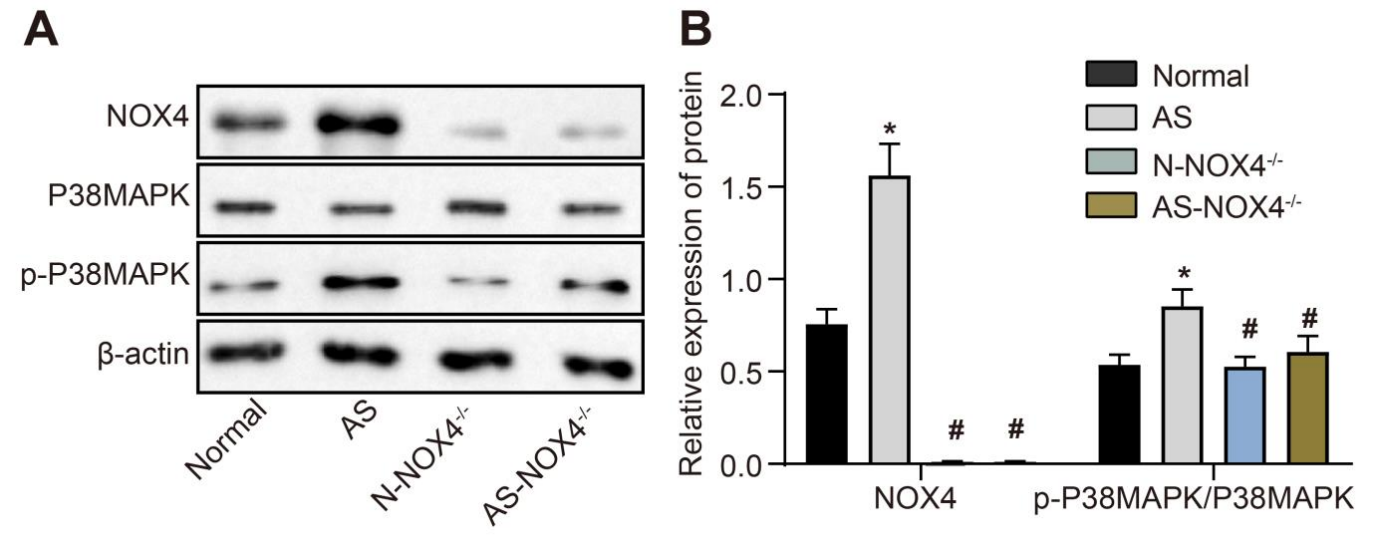
**The protein expression of NOX4 and ratio of p-p38 MAPK/p38 MAPK are upregulated in coronary artery tissues of AS mice.** (**A**) The protein expressions of NOX4, p38 MAPK and p-p38 MAPK were detected by Western blot; (**B**) Statistical comparison of NOX4, p38 MAPK and p-p38 MAPK protein expressions among four groups; The data were analyzed by one-way ANOVA with Tukey's test; n = 10; * p < 0.05 vs. the normal group; # p < 0.05 vs. the AS group; AS, atherosclerosis; NOX4, NADPH oxidase 4.

Figure 2A, 2B depicts the protein expression of NOX4 and p38 MAPK and the extent of p38 MAPK phosphorylation in the coronary artery tissues of the normal, AS, N-*NOX4*^-/-^ and AS-*NOX4*^-/-^ groups, as detected by Western blot analysis. The protein expression of NOX4 and the ratio of p-p38 MAPK/p38 MAPK were upregulated in coronary artery tissues of the AS group compared to the normal group (p < 0.05). No protein expression of NOX4 was observed in the coronary artery tissues of the N-*NOX4*^-/-^ and AS-*NOX4*^-/-^ groups (p > 0.05), whereas the ratio of p-p38 MAPK/p38 MAPK was decreased compared with the AS group. These results suggested that the increased p-p38 MAPK/p38 MAPK by AS could be inhibited by the *NOX4* knockout.

### Differential expression of miR-363-3p, *NOX4*, and p38 MAPK in homocysteine (HCY)-induced coronary arterial endothelial cells (CAECs)

**Figure 3:**
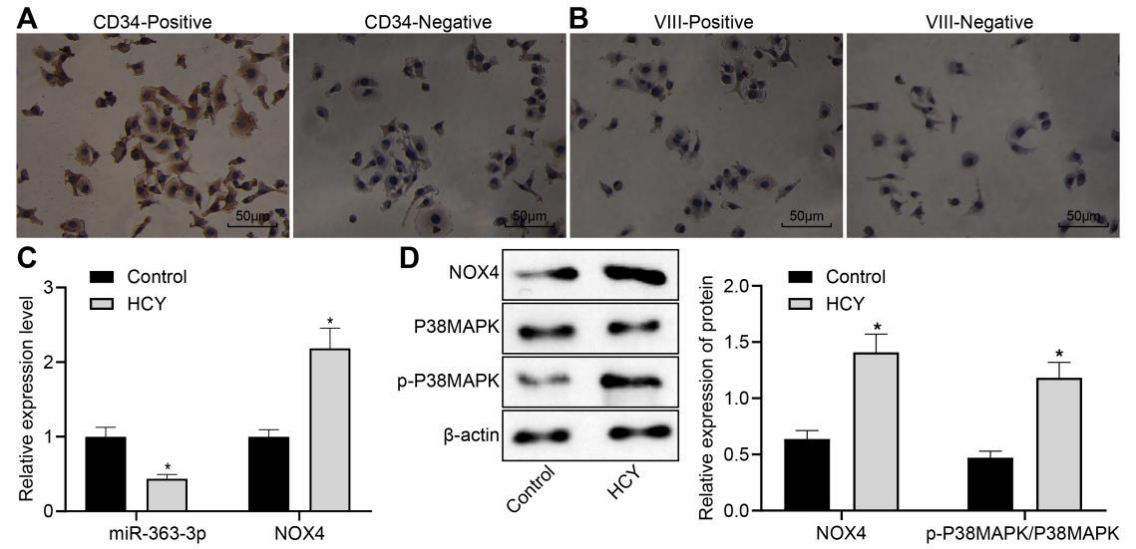
**Reduced miR-363-3p expression and abundant *NOX4* and p38 MAPK expressions in HCY-induced CAECs.** (**A**) positive and negative staining for goat antibody to CD34 (scale bar = 25 μm); (**B**) positive and negative staining for rabbit antibody to Factor VIII (scale bar = 25 μm); (**C**) miR-363-3p expression and *NOX4* mRNA expression were determined by RT-qPCR in HCY-induced CAECs; (**D**) Representative Western blots of *NOX4*, p38 MAPK and p-p38 MAPK proteins and their quantitation in HCY-induced CAECs, normalized by β-actin. The data were analyzed by paired t-test. n = 3. * p < 0.05 vs. the control group; CAECs, coronary arterial endothelial cells; SP, streptavidin-peroxidase.

We isolated primary CAECs from normal mice and used a goat antibody to CD34 for immunohistochemical detection, which clearly demonstrated brown staining in the cytoplasm of the positive staining group but no brown staining in the negative control (NC) group. Immunohistochemical staining with a rabbit antibody to factor VIII was negative in the positive- and negative-staining groups, thereby confirming that the isolated cells were indeed endothelial cells. These results are shown in Figure 3A, 3B. Next, we investigated the role of the miR-363-3p/*NOX4*/p38 MAPK axis in CAECs. HCY was incubated with the isolated CAECs for 24 h for CHD induction. The results of the RT-qPCR showed a downregulated miR-363-3p expression and an upregulated mRNA expression of *NOX4* in the CAECs in the HCY group in comparison to the control group (p < 0.05, Figure 3C). Moreover, as illustrated in Figure 3D, the protein expression of NOX4 and the ratio of p-p38 MAPK/p38 MAPK were augmented in the CAECs in the HCY group compared to the control group (p < 0.05). These findings suggested a reduced miR-363-3p and elevated *NOX4* and p38 MAPK in HCY-induced CAECs.

### *NOX4* is a target gene of miR-363-3p

**Figure 4:**
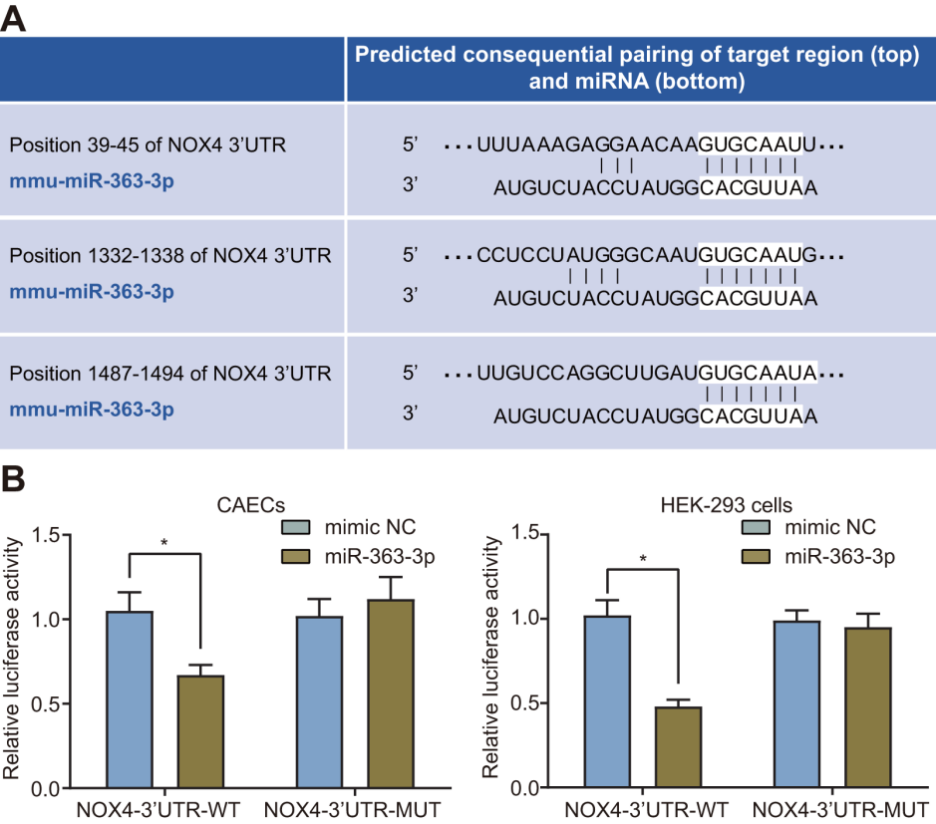
**miR-363-3p binds to the 3’UTR *NOX4* mRNA and inhibits its expression in CAECs and HEK-293 cells.** (**A**) putative miR-363-3p binding sites in the 3’UTR of *NOX4* mRNA in the online website (http://www.targetscan.org); (**B**) miR-363-3p binding with the 3’UTR of *NOX4* mRNA in CAECs and HEK-293 cells confirmed by dual luciferase reporter assay. Comparisons between two groups were performed by unpaired t-test, and t-test was adopted for pairwise comparisons. n = 3; * p < 0.05 vs. the mimic NC group; miR, microRNA; *NOX4*, NADPH oxidase 4; 3’UTR, 3’ untranslated region.

The target relationship between miR-363-3p and *NOX4* was predicted using the website available at http://www.targetscan.org (Figure 4A). Dual luciferase reporter assay was further conducted to validate the predicted relationship, which showed a reduced luciferase activity in the *NOX4*-3’untranslated region (3’UTR)-wild type (WT) (p < 0.05) but no significant difference in the luciferase activity of the *NOX4*-3’UTR-mutant (MUT) in the CAECs and the HEK-293 cells transfected with miR-363-3p mimic (p > 0.05) (Figure 4B). Therefore, miR-363-3p could target *NOX4* and subsequently downregulated its expression in CAECs and HEK-293 cells.

### Selection of an optimal small interfering RNA (siRNA) against *NOX4* in CAECs

RT-qPCR was conducted for the detection of the mRNA expression of *NOX4* to select the siRNA plasmid with the optimal silencing rate. In comparison with the NC group, the mRNA expression of *NOX4* was lower in the siRNA *NOX4*-1, siRNA *NOX4*-2 and siRNA *NOX4*-3 groups (p < 0.05), with the siRNA *NOX4*-1 group showing the highest silencing efficiency (p < 0.05). No significant difference was observed between the NC group and the normal group (p > 0.05) (Table 3). Hence, siRNA *NOX4*-1 was selected for subsequent experimentation.

**Table 3:** The siRNA *NOX4*-1 group exhibited a highest silencing efficiency.

**Group**	**NOX4 mRNA**	**Silencing rate (%)**
siRNA *NOX4*-1	0.246 ± 0.033^*#^	75.79 ± 3.25
siRNA *NOX4*-2	0.467 ± 0.011^*#&^	33.87 ± 1.75
siRNA *NOX4*-3	0.492 ± 0.023^*#&^	31.15 ± 2.33
Negative control	0.816 ± 0.012	3.43 ± 1.24
Blank control	0.845 ± 0.042	

Note: The data were analyzed by one-way ANOVA with Tukey's test; n = 3; * *p* < 0.05 *vs.* the negative control group; ^#^ *p* < 0.05 *vs.* the blank control group; ^&^ *p* < 0.05 *vs.* the siRNA *NOX4*-1 group; *NOX4*, NADPH oxidase 4.

### MiR-363-3p negatively regulates *NOX4* and blocks the p38 MAPK signaling pathway in CAECs

**Figure 5:**
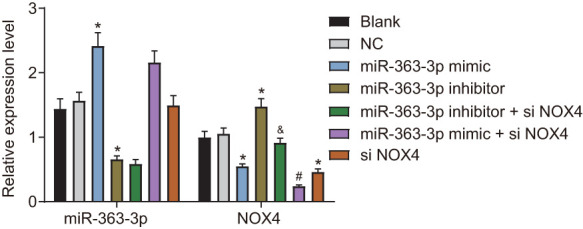
**miR-363-3p downregulates the mRNA expression of *NOX4* in CAECs.** The data were analyzed by one-way ANOVA with Tukey's post hoc test; n = 3; * *p* < 0.05 vs. the NC group; # *p* < 0.05 vs. the miR-363-3p mimic group; & p < 0.05 vs. the miR-363-3p inhibitor group; CAECs, coronary artery endothelial cells; miR, microRNA; *NOX4*, NADPH oxidase 4; NC, negative control.

After uncovering the regulatory relationship between miR-363-3p and *NOX4*, we next aimed to elucidate the possible downstream mechanism of *NOX4* in CAECs. First, we stably expressed miR-363-3p mimic, miR-363-3p inhibitor, and siNOX4, either separately or in combination, in CAECs and incubated the cells with HCY for 24 h for stress-induced CHD. Next, the expression of *NOX4* in CAECs was determined by RT-qPCR (Figure 5), which showed that the mRNA expression of *NOX4* was reduced in the miR-363-3p mimic group and the siNOX4 group, while it was higher in the miR-363-3p inhibitor group, compared to the NC group (p < 0.05). In comparison with the miR-363-3p inhibitor group, mRNA expression of *NOX4* was reduced in the miR-363-3p inhibitor + si*NOX4* group (p < 0.05). The mRNA expression of *NOX4* was even lower in the miR-363-3p mimic + siNOX4 group than in the miR-363-3p mimic group (p < 0.05). These results indicated that miR-363-3p extensively suppressed the expression of *NOX4* in CAECs.

**Figure 6:**
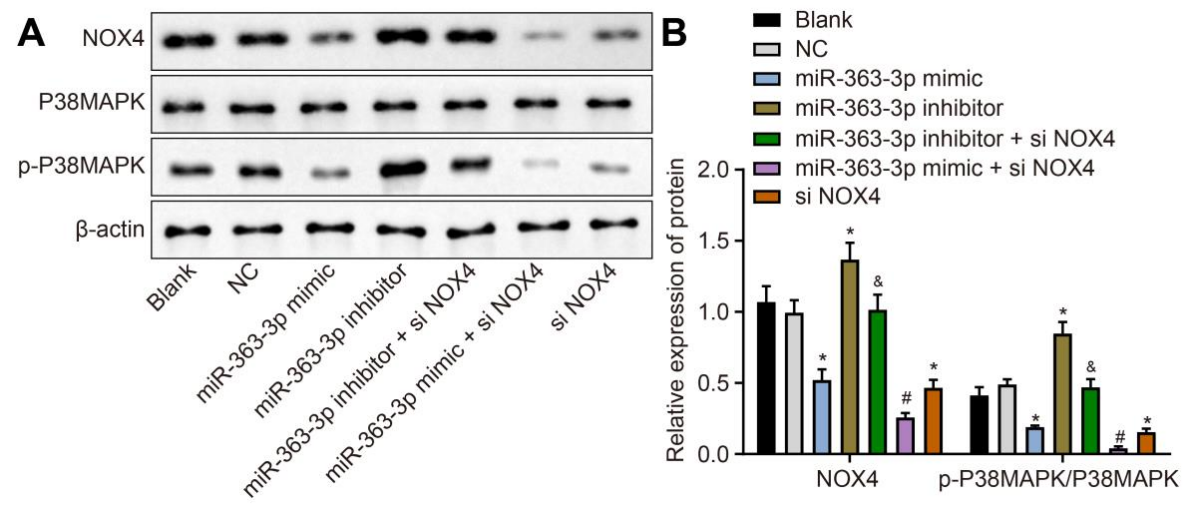
**miR-363-3p downregulates protein expression of *NOX4* and the ratio of p-p38 MAPK/p38 MAPK in CAECs.** (**A**, **B**) Representative Western blots of *NOX4*, p38 MAPK and p-p38 MAPK proteins and their quantitation in CAECs, normalized by β-actin; the data were analyzed by one-way ANOVA with Tukey's post hoc test; n = 3; * p < 0.05 vs. the blank group; # p < 0.05 vs. the miR-363-3p mimic group; & p < 0.05 vs. the miR-363-3p inhibitor group; CAECs, coronary artery endothelial cells; miR, microRNA; *NOX4*, NADPH oxidase 4; NC, negative control.

Western blot analysis (Figure 6A, 6B) showed a lower NOX4 protein expression and p-p38 MAPK/p38 MAPK ratio in the miR-363-3p mimic and the siNOX4 groups than in the NC group (p < 0.05). However, these markers were increased in the miR-363-3p inhibitor group compared to the NC group (p < 0.05). The protein expression of NOX4 and the ratio of p-p38 MAPK/p38 MAPK were lower in the miR-363-3p inhibitor + siNOX4 group than in the miR-363-3p inhibitor group (p < 0.05). In comparison with the miR-363-3p mimic group, the miR-363-3p mimic + siNOX4 group showed a lower NOX4 protein expression, along with a reduced ratio of p-p38 MAPK/p38 MAPK (p < 0.05). Taken together, these data uncovered that miR-363-3p downregulated *NOX4* and blocked the activation of the p38 MAPK signaling pathway in CAECs.

### miR-363-3p promotes CAEC viability by targeting *NOX4*

**Figure 7:**
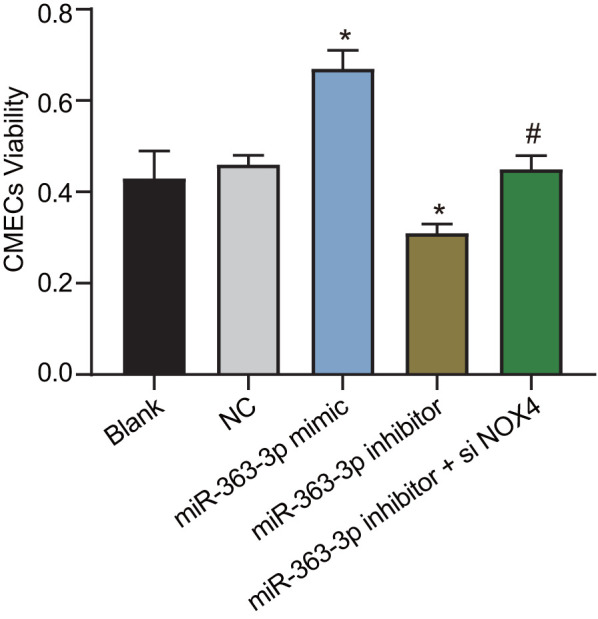
**miR-363-3p promotes CAEC viability by targeting *NOX4*.** The data were analyzed by one-way ANOVA with Tukey's post hoc test; n = 3; * p < 0.05 vs. the NC group; # p < 0.05 vs. the miR-363-3p inhibitor group; CAECs, coronary artery endothelial cells; miR, microRNA; *NOX4*, NADPH oxidase 4; NC, negative control.

Figure 7 shows the viability of CAECs as measured by the 3-(4,5-dimethylthiazol-2-yl)-2,5-diphenyltetrazolium bromide (MTT) assay. In comparison with the NC group, the cell viability was increased in the miR-363-3p mimic group, while it was lower in the miR-363-3p inhibitor group (p < 0.05). When compared to the miR-363-3p inhibitor group, the cell viability was increased in the miR-363-3p inhibitor + siNOX4 group (p < 0.05). These results highlighted the potential of miR-363-3p to increase the viability of CAECs by targeting *NOX4*.

### miR-363-3p reduces the hydrogen peroxide (H_2_O_2_) level and catalase (CAT) activity in CAECs by targeting *NOX4*

**Figure 8:**
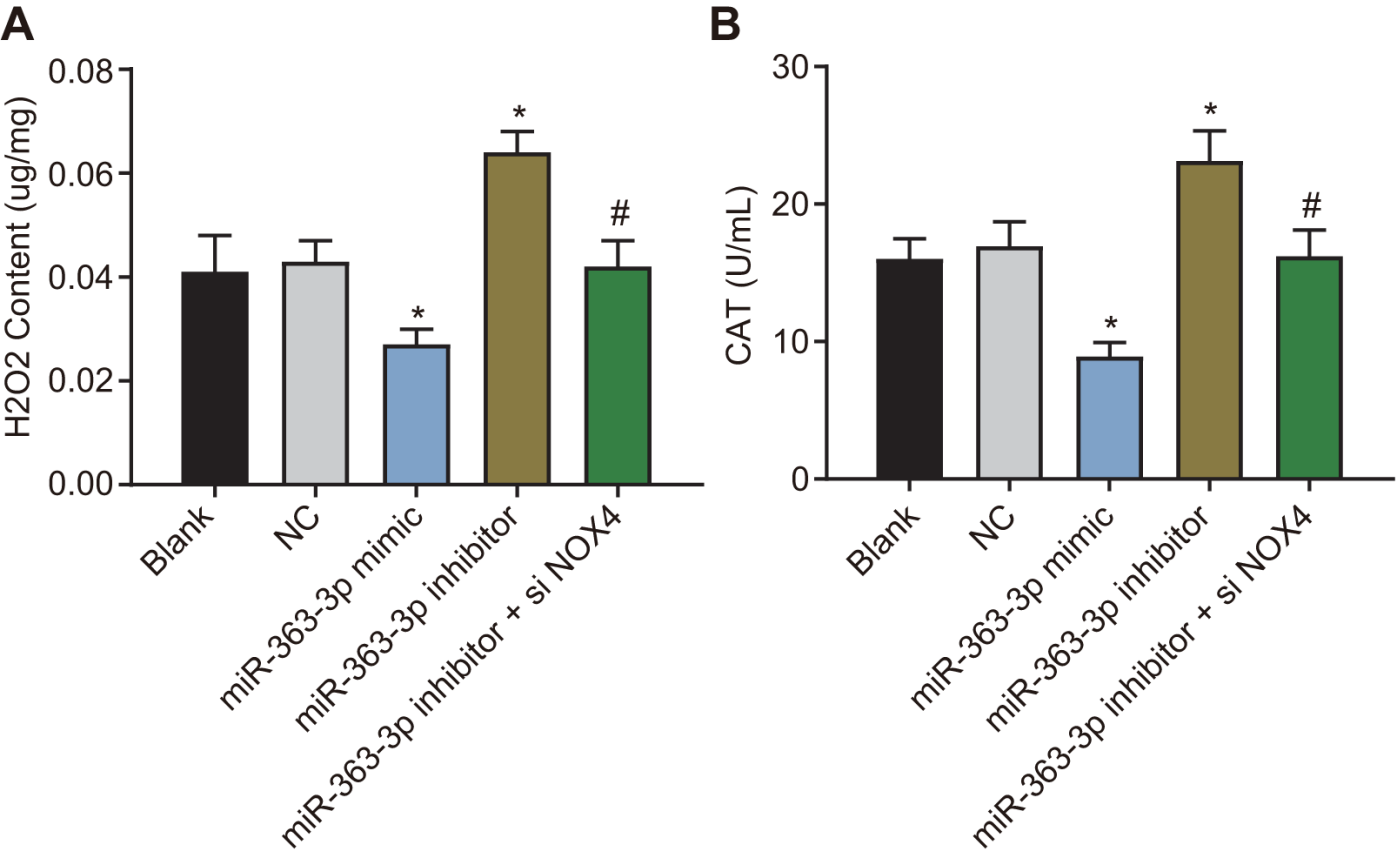
**miR-363-3p reduces H_2_O_2_ level and CAT activity in CAECs by targeting *NOX4*.** (**A**) quantification of H_2_O_2_ level in CAECs; (**B**) quantification of CAT activity in CAECs; the data were analyzed by one-way ANOVA with Tukey's post hoc test; n = 3; * p < 0.05 vs. the blank group; # p < 0.05 vs. the miR-363-3p mimic group; CAECs, coronary artery endothelial cells; CAT, catalase; miR, microRNA; *NOX4*, NADPH oxidase 4; NC, negative control.

H_2_O_2_ is an important active molecule that mediates oxidative stress-induced endothelial injury, and *NOX4* is the major producer of H_2_O_2_ in living cells [22]. CAT is a kind of antioxidant enzyme widely distributed in almost all organisms, the main function of which is to catalyze H_2_O_2_ into water and O_2_ and to clear the H_2_O_2_ in the body to protect the cells from the toxicity of H_2_O_2_. The CAT level can also reflect the oxidative stress [23]. Therefore, we next examined the effect of miR-363-3p on the H_2_O_2_ level and the CAT activity in CAECs. In comparison with the NC group, the H_2_O_2_ level and CAT activity were decreased in the miR-363-3p mimic group, which were increased in the miR-363-3p inhibitor group (p < 0.05). The levels of H_2_O_2_ and CAT activity were much lower in the miR-363-3p inhibitor + siNOX4 group than in the miR-363-3p inhibitor group (p < 0.05) (Figure 8A, 8B). These results indicated that miR-363-3p could reduce the H_2_O_2_ levels and CAT activity in CAECs by targeting *NOX4*.

### miR-363-3p inhibits apoptosis in CAECs by targeting *NOX4*

**Figure 9:**
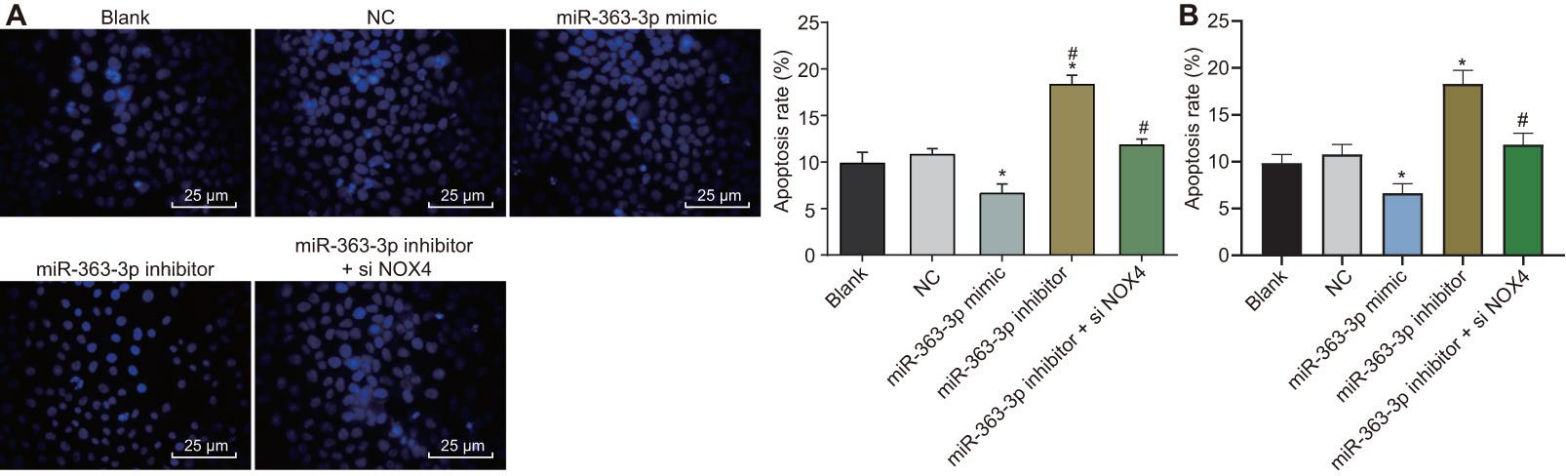
**miR-363-3p attenuates apoptosis of CAECs by targeting *NOX4*.** (**A**) Hoechst 33258 staining and quantification of apoptosis in CAECs (scale bar = 25 μm); (**B**) flow cytometry and quantification of apoptosis in CAECs; * p < 0.05 vs. the blank group; # p < 0.05 vs. the miR-363-3p inhibitor group; the data were analyzed by one-way ANOVA with Tukey's post hoc test; n = 3; CAECs, coronary artery endothelial cells; miR, microRNA; *NOX4*, NADPH oxidase 4; NC, negative control.

Hoechst 33258 staining and flow cytometry were employed to observe the apoptosis in CAECs, as shown in Figure 9A, 9B. No difference was evident in the apoptosis rate between the blank group and the NC group (p > 0.05). In comparison with the NC group, the apoptosis rate was decreased in the miR-363-3p mimic group and increased in the miR-363-3p inhibitor group (p < 0.05). When compared with the miR-363-3p inhibitor group, the apoptosis rate was attenuated in the miR-363-3p inhibitor + siNOX4 group (p < 0.05). According to these results, the apoptosis of CAECs could be repressed by miR-363-3p through inhibition of *NOX4*.

### miR-363-3p increases tube formation of CAECs by targeting *NOX4*

**Figure 10:**
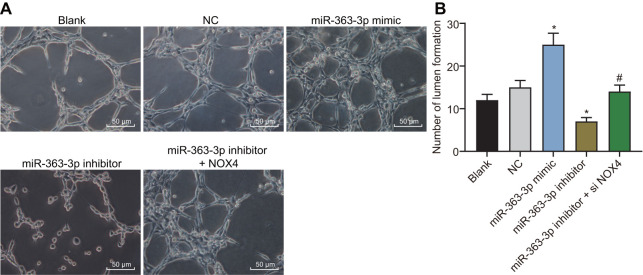
**miR-363-3p enhances tube formation of CAECs by targeting *NOX4*.** (**A**) representative images of the tubular structures in CAECs (scale bar = 100 μm); (**B**) quantification of tubular structures in CAECs; the data were analyzed by one-way ANOVA with Tukey's test; n = 3; * p < 0.05 vs. the blank group; # p < 0.05 vs. the miR-363-3p mimic group; CAECs, coronary artery endothelial cells; miR, microRNA; *NOX4*, NADPH oxidase 4; NC, negative control.

The tube formation assay was performed to examine the effect of miR-363-3p on the tube formation of CAECs. As depicted in Figure 10A, 10B, the number of tubular structures was increased in the miR-363-3p mimic group and reduced in the miR-363-3p inhibitor group compared with the NC group (p < 0.05). In comparison with the miR-363-3p inhibitor group, the number of tubular structures formed in the miR-363-3p inhibitor + siNOX4 group was increased (p < 0.05). These results indicated that miR-363-3p stimulated tube formation in CAECs through inhibition of *NOX4*.

### miR-363-3p reduces the expression of inflammatory factors in CAECs by targeting *NOX4*

Enzyme-linked immunosorbent assay (ELISA) was conducted to observe the effects of miR-363-3p on inflammatory responses in CAECs. In comparison to the NC group, the secretion of interleukin (IL)-6, intercellular adhesion molecule-1 (ICAM-1), and IL-1β was lower in the miR-363-3p mimic group, accompanied by an increased IL-10 secretion (p < 0.05). In contrast, the miR-363-3p inhibitor group showed increased secretion of IL-6, ICAM-1, and IL-1β, as well as a lower secretion of IL-10 (p < 0.05). Relative to the miR-363-3p inhibitor group, the miR-363-3p inhibitor + siNOX4 group displayed reduced secretion of IL-6, ICAM-1 and IL-1β, along with elevated secretion of IL-10 (p < 0.05) (Table 4). The aforementioned data indicated that miR-363-3p could inhibit the expression of proinflammatory cytokines in CAECs through inhibition of *NOX4*.

**Table 4:** Levels of the IL-6, ICAM-1 and IL-1β were increased while IL-10 level was reduced in cells treated with miR-363-3p inhibitor (pg/mL).

**Group**	**IL-6**	**ICAM-1**	**IL-10**	**IL-1β**
Blank	49.26 ± 5.02	24.57 ± 7.65	48.56 ± 11.05	89.14 ± 6.48
Negative control	51.64 ± 5.50	27.45 ± 8.19	50.43 ± 12.61	95.25 ± 4.64
miR-363-3p mimics	27.55 ± 3.31^*^	12.34 ± 6.31^*^	82.26 ± 16.53^*^	45.88 ± 3.89^*^
miR-363-3p inhibitors	89.56 ± 7.58^*^	42.64 ± 13.02^*^	25.96 ± 8.37^*^	188.28 ± 5.31^*^
miR-363-3p inhibitors + si *NOX4*	53.61 ± 5.34^#^	25.41 ± 6.18^#^	46.13 ± 13.12^#^	91.57 ± 4.25^#^

Note: The data were analyzed by one-way ANOVA with Tukey's test; n = 3; * *p* < 0.05 *vs.* the blank group; ^#^ *p* < 0.05 compared with the miR-363-3p mimics group; IL-6, interleukin-6; ICAM-1, intercellular cell adhesion molecule-1; IL-10, interleukin-10; IL-1β, interleukin-1β; *NOX4*, NADPH oxidase 4.

## DISCUSSION

This study was designed to investigate the effects of miR-363-3p on endothelial dysfunction and the inflammatory response in CHD based on a hypothesis involving molecular targeting of *NOX4* via the p38 MAPK signaling pathway. Our findings suggested that miR-363-3p impeded the apoptosis in CAECs and the expression of pro-inflammatory cytokines in AS mice with CHD by inhibiting the *NOX4*-dependent p38 MAPK signaling pathway.

Our initial findings highlighted that AS mice had elevated serum levels of TC, TG, LDLC and calcium compared with the normal mice. In the clinic, an elevated level of LDLC is considered as a major risk factor for CHD, and reductions in the levels of TC, TG and LDLC are associated with a reduced morbidity and mortality of AS [24]. Consistent with our present results, Prendergast et al. has reported the presence of elevated calcium levels in endothelial cells prior to plaque development [25].

In the present study, we found a low expression of miR-363-3p but a high expression of *NOX4* in both the coronary artery tissues of AS mice and the HCY-induced CAECs. miR-363-3p, a member of the miR-363 family, is derived from the miR-106a-363 cluster [26]. The miR-106a-363 cluster is a paralog of the extensively studied miR-17-92 cluster, which has been linked to the cardiovascular system [27, 28]. Previous studies also demonstrated that miR-363 is dysregulated in several cancers and is specifically downregulated in numerous cancers, such as head and neck squamous cell carcinoma and hepatocellular carcinoma [29, 30]. miR-363-3p suppresses metastasis and inhibits the epithelial-to-mesenchymal transition (EMT) in colorectal cancer by targeting Sox4 [31]. Interestingly, miR-363 was reported to negatively regulate HAND1, a vital left ventricular determining transcription factor, thereby functioning as a modulator in the generation of functional left-ventricular cardiomyocytes [11]. *NOX4* has been found to be extensively expressed in H_2_O_2_-induced human coronary artery smooth muscle cells in AS [32]. There was a trend toward an increased expression of *NOX4* in oxygen and glucose depletion/reoxygenation (OGD/R)-treated human brain vascular endothelial cells collected after AS-associated stroke [33]. Upregulation of *NOX4* is evident in AS endothelial cells, thereby highlighting the vital functionality of the *NOX4*-containing NADPH oxidase [34].

miRNAs can modulate gene expression posttranscriptionally by interacting with the 3’UTR of specific target mRNAs [35]. In this study, a biological prediction website and a luciferase reporter assay identified that miR-363-3p bound to the 3’UTR of *NOX4* mRNA and could negatively regulate its expression. A previous study revealed that miR-363-3p is involved in the metastasis of colorectal cancer by functioning as a tumor suppressor through the negative regulation of its target Sox4 gene [31]. MiR-363-3p can also function as a tumor suppressor in papillary thyroid carcinoma, where its suppressive effect is mediated by repression of PIK3CA [36]. The aforementioned studies collectively demonstrate that miR-363-3p can target different genes to affect the development of disease in several types of carcinomas.

*NOX4*, as a key cellular regulator of oxidative stress [37], is associated with the involvement of miR-363-3p in AS. H_2_O_2_ is considered as a key active molecule mediating the oxidative stress-induced endothelial injury in AS, and *NOX4* has been found to be the major producer of H_2_O_2_ [22]. *NOX4* knockdown has also been documented to elicit a decrease of H_2_O_2_ levels in human umbilical vein endothelial cells (HUVECs) treated with copper oxide nanoparticles (CuONPs), which normally evoke oxidative stress [38]. Treatment of cyclosporine A rats with CAT, an H_2_O_2_ scavenger, is capable of restoring *NOX4* expression [39]. Endothelial dysfunction in the ascending aorta in a Marfan’s disease model has been prevented in mice following the knockdown of *NOX4* [40]. Bao et al. confirmed that oxidative stress could influence p38 MAPK signaling and various downstream transcription factors, resulting in alterations in gene expression capable of impacting cell survival. Other reports have highlighted that these alterations involve reduced oxidative stress via *NOX4* and regulation of p38 MAPK [18]. Another study reported a significant decrease in p38 MAPK mRNA at two days after transfection with the miRNA mimics [41]. Moreover, the multiMiR package in R showed that hsa-miR-363-3p was downregulated in gastric cancer and significantly enriched in the MAPK signaling pathway [42]. It is well known that MAPKs include ERK1/2, ERK5, p38 and JNK [43]. siRNA-mediated *NOX4* silencing reduces ROS generation, p38 expression and ERK1/2 phosphorylation level in HUVECs, thus retarding TNF-α-induced oxidative stress and inflammation in AS [44]. In palmitic acid (PA)-induced murine hepatic AML12 cells, treatment with the JNK inhibitor SP600125 decreases *NOX4* expression [45]. Inhibition of *NOX4* and inactivation of JNK helped to mitigate the inflammatory response in fibroblast-like synoviocytes, exerting protective effects against the progression of rheumatoid arthritis [46]. Although there is no literature establishing a direct relationship between *NOX4* and ERK5 (also named BMK1), in the aldosterone or high salt-induced hypertensive renal injury rat model, the expression of the *NOX4* gene, along with ERK1/2, JNK and BMK1, is all increased, but the p38 MAPK activity was unchanged [47]. The aforementioned results revealed the close relationship between *NOX4* and the MAPK members, along with their complex regulatory relationship. Thus, we cannot exclude the possibility of involvement of other signaling pathways in the pro-inflammatory effects of *NOX4* in CAECs, given the very complex cellular microenvironments and the capacity of p38 MAPK to interact with other signaling pathways. The interaction between *NOX4* and the p38 MAPK signaling pathway merits further in-depth analysis.

Furthermore, the present study demonstrated that miR-363-3p increased CAEC viability, tube formation, and IL-10 levels, as well as reducing apoptosis in the CAECs and the expression of the pro-inflammatory cytokines IL-6, ICAM-1, and IL-1β. These findings are indicative of the ability of miR-363-3p to suppress inflammation, thereby preventing endothelial cell injury. Inhibition or overexpression of certain miRs represents a promising therapeutic channel for the treatment of cardiovascular disorders [7]. Another study also found that reduced miR-363 expression resulted in a significantly decreased number of newly formed tubes among endothelial cells [13]. Furthermore, miR-363-3p suppresses the proliferation of human hepatocellular carcinoma cells by targeting S1PR1 or USP28 [48]. This phenomenon has also been evident in head and neck squamous cell carcinomas, where miR-363-3p inhibits the expression of the transmembrane glycoprotein podoplanin [29]. Cytokines, by functioning as modulators of immune responses and pro-inflammatory and anti-inflammatory stimulation, may play a significant role in the development of AS [49]. Although previous studies have characterized the relationship between miR-17-92 and immune responses [50], an understanding of the detailed mechanisms of how miRs affect specific cytokines still requires further investigation.

**Figure 11:**
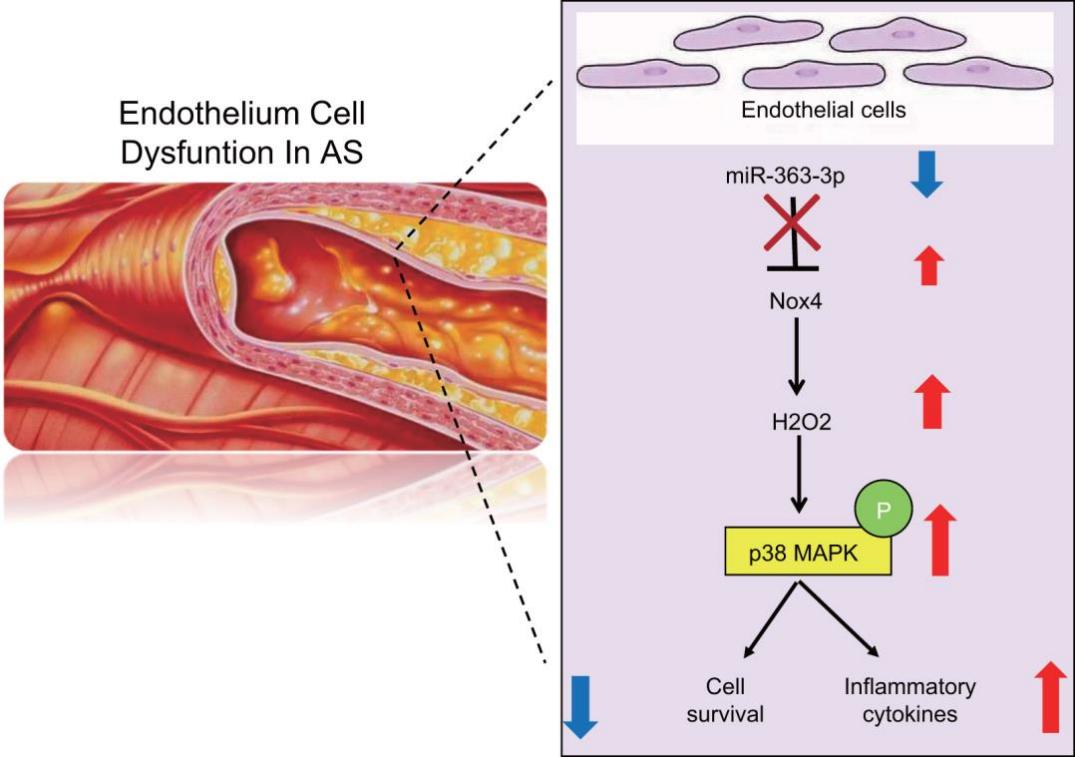
**A schematic for the function and mechanism of miR-363-3p in CHD.** miR-363-3p targets *NOX4* and disrupts the p38 MAPK/ signaling pathway, which consequently reduces the apoptosis of CAECs, oxidative stress injury and the expression of inflammatory factors in CAECs, thus protecting CAECs against CHD.

Overall, this study suggests that miR-363-3p confers cellular protection against inflammation-induced endothelial cell injury in an AS model by targeting *NOX4* via inactivation of the p38 MAPK signaling pathway (Figure 11). The results of this study provide novel insights for the development of potential therapeutic strategies in the treatment of CHD. Further comprehensive experiments are required to expand our observations and to explore the mechanism of miR-363-3p-mediated downregulation of *NOX4* and the relationships between miR-363-3p and specific inflammatory cytokines.

## MATERIALS AND METHODS

### Ethics statement

This study was conducted with the approval of the Ethics Committee of the Guizhou Provincial People’s Hospital and in strict accordance with the Guide for the Care and Use of Laboratory Animals published by the US National Institutes of Health. Adequate measures were taken to minimize the numbers of animals and their suffering.

### Study subjects

Twenty healthy male C57BL/6 mice (aged 3-4 months; weighing 25 ± 5 g) were acquired from the Peking University Health Science Center (Beijing, China). These mice were housed in plastic cages with a stainless-steel bottom, with five mice in each cage. Twenty healthy male *NOX4*^-/-^ clean mice with a C57BL/6 genetic background were provided by the Peking University Health Science Center (Beijing, China). These mice were housed in the Laboratory of Clinical Medicine, which conformed to the grade 2 animal breeding standards. All mice were given ad libitum access to food and water.

### Animal grouping and model establishment

The mice were assigned into the normal group (10 normal mice), the AS group (10 mice with AS), the N-*NOX4*^-/-^ group (10 *NOX4*^-/-^ normal mice) and the AS-*NOX4*^-/-^ group (10 *NOX4*^-/-^ mice with AS).

The AS mouse model was established in strict accordance with the procedure described by Fu et al. [51]. After a week of adaptive feeding, the mice in the normal and *NOX4*^-/-^ groups were fed with conventional food and tap water, whereas mice in the AS and AS-*NOX4*^-/-^ groups were fed with vitamin D3 powder at a concentration of 1.25 × 10^6^ μg/kg along with a high-fat diet comprised of cholesterol (2%), sodium cholate (0.5%), lard (3%), propylthiouracil (0.2%) and basic food (94.3%). At the beginning of the experiment, the mice were given an intramuscular injection of vitamin D3 powder at a dose of 3 × 10^6^ μg/kg in the right lower extremity. This injection protocol was repeated once every 30 days.

### Collection of arterial blood and coronary artery specimens

Three months after model establishment, arterial blood and coronary artery specimens were obtained from mice in each group after overnight fasting. Under deep anesthesia via an intraperitoneal injection of sodium pentobarbital, the abdominal aorta was isolated and opened below the renal artery bifurcation. A puncture needle was then inserted, and an arterial blood sample was withdrawn using a syringe and centrifuged (3000 rpm) at 4° C for 5 min. Next, 1 mL of the separated serum was collected for determination of blood lipid and calcium levels. Additionally, a segment of the coronary artery (approximately 1 cm in length) was excised from the lower border of the coronary artery. The segment was fixed using 4% paraformaldehyde, embedded in paraffin and sliced into serial sections.

### Detection of blood lipid and calcium levels

An Abbott Aeroset^TM^ biochemical analyzer (Abbott Laboratories, Abbott Park, IL, USA) was utilized to measure the serum levels of TC, TG, HDLC, LDLC and calcium in the mice.

### Movat’s pentachromic staining

The paraffin-embedded coronary artery tissues were sectioned, dewaxed using xylene, and then treated with 5% sodium thiosulfate and 1% Alcian blue solution for 20 min. The sections were then heated at a high temperature in a microwave for 45 s in alkaline ethanol. Next, the sections were stained using Weigert hematoxylin solution, saffron/acid fuchsin solution and 5% phosphotungstic acid solution sequentially for 5 min each. Following the final staining regimen, the sections were transferred into 1% glacial acetic acid for 5 min. After conventional gradient ethanol dehydration, the sections were cleared using xylene, mounted using neutral gum and observed under a Leica optical microscope. The plaque area and vascular area were then measured and documented.

### Immunohistochemical staining

The coronary artery tissues of mice in each group were resected and fixed in a conventional manner using 4% paraformaldehyde for 24 h, followed by dehydration with an ethanol series (80%, 90%, and 100%) and n-butanol. Next, the sections were immersed in a 60° C wax box, embedded, and then sliced into 5-μm serial sections. The sections were subsequently heated for 1 h at 60° C, dewaxed with xylene, rehydrated with gradient ethanol, and soaked in 3% H_2_O_2_ for 10 min, followed by high-pressure antigen retrieval for 90 s. The sections were then blocked with 100 μL of 5% bovine serum albumin (BSA) blocking solution and later incubated at 37° C for 30 min. The sections were incubated with 100 μL of the primary rabbit antibody against CD34 (1:1000, ab214035, Abcam, Cambridge, UK) at 4° C overnight. The sections were then incubated with the biotin-labeled secondary antibody, goat anti-rabbit working solution (1:100; HY90046, Shanghai HengYuan Biological Technology Co., Ltd., Shanghai, China), at 37° C for 30 min. After that, the sections were incubated in streptavidin-peroxidase solution (Beijing Zhongshan Biotechnology Co., Ltd., Beijing, China) at 37° C for 30 min and finally treated with diaminobenzidine (DAB) (Beijing Bioss Biotechnology Co., Ltd., Beijing, China) for development at room temperature. The sections were then soaked in hematoxylin for 5 min, rinsed in 1% hydrochloric acid for 4 s, and then immersed in tap water for 20 min to revert to the original blue color. The criterion for CD34 protein-positive cells was the appearance of a brown yellow color as observed by a microscopy. The Image-Pro Plus image analysis software (Media Cybernetics, Silver Springs, MD, USA) was used to detect the mean optical density (OD) values of the CD34-positive coloration in the coronary tissues of each group under high magnification microscopy, followed by a quantitative analysis.

### Culture and identification of CAECs

The proximal coronary artery of normal mice was excised and soaked in a culture dish containing phosphate-buffered saline (PBS) and heparin for approximately 5 min to prevent any subsequent blood coagulation. The coronary artery was then rinsed 3 times with sterile PBS and soaked in 75% ethanol for 15 s. The artery tissues were sliced into 1 mm^3^ tissue blocks using a pair of ophthalmic scissors and detached for 6 min at 37° C using 3 mL of 0.2% type II collagenase. Subsequently, an equal volume of 0.25% trypsin was added, and the tissues were placed in a water bath at 37° C for 5 min, after which the detachment was terminated by the addition of 3 mL of Dulbecco's modified Eagle's medium (DMEM) containing 10% fetal bovine serum (FBS). After the supernatant was discarded, the cells were resuspended in complete culture medium and incubated with 5% CO_2_ at 37° C. After 6 h, the medium was replaced, and the non-adherent cells were removed. When the remaining cells reached 80% confluence, the medium was discarded. Then, solutions of 0.25% trypsin and 0.02% ethylenediamine tetra acetic acid (EDTA) were added for complete detachment under gentle shaking, followed by microscopic observation. After contraction and conversion to a round shape by the majority of cells in the culture, a medium containing 10% FBS was added to terminate the detachment. The cells were then triturated using a pipette for detachment, and the cell suspension was centrifuged and resuspended in complete culture medium. After adjustment of the cell density to an appropriate level, the cells were maintained and passaged at 37° C with 5% CO_2_. The culture medium was substituted once every 3 days.

Streptavidin-peroxidase (SP) immunohistochemical staining was performed to identify the CAECs in mice; rabbit antibody to factor VIII and goat antibody to CD34 were selected as the primary antibodies. When the CAECs reached 80% confluence, they were detached by adding trypsin and then seeded onto coverslips in a 6-well plate. After being seeded with the CAECs, the coverslips were fixed using paraformaldehyde for 30 min and incubated with the specified diluted primary antibodies (PBS used as the NC) overnight at 4° C. The CAECs were then incubated with the goat anti-rabbit IgG at 37° C for 40 min. After that, the CAECs were developed with DAB, stained using hematoxylin for 1 min, differentiated using hydrochloric acid for 2 s, sealed with conventional resin and observed under an inverted microscope.

### Plasmid construction of siRNA against *NOX4*

The *NOX4* gene sequences were obtained from the GenBank. In all, 3 pairs of siRNA sequences were designed in strict accordance with the basic siRNA design principles provided online by Ambion Inc. (Austin, TX, USA). Furthermore, a BLAST analysis was then performed to determine the specificity of the siRNAs. The siRNAs against *NOX4* were constructed and identified by the Shanghai Genechem Co., Ltd. (Shanghai, China). The gene sequences are shown in Table 5.

**Table 5:** The siRNA primer sequences.

**Gene**	**Sequence**
siRNA *NOX4*-1	F: 5'-CAUGCUGCUGCUGUUGCAUGUUUCA-3' R: 5'-CCCUCUGAUGUAAUGGAACUCCGUA-3'
siRNA *NOX4*-2	F: 5′-GACCUGACUUUGUGAACAUTT-3′ R: 5′-AUGUUCACAAAAUCAGGUCTT-3′
siRNA *NOX4*-3	F: 5′-GCTGTGATAAUCACCUCUAC-3′ R: 5′-GATTGAGGUGAUUAUCACAGC-3′
*NOX4*	F: 5’-TAGCTGCCCACTTGGTGAACG-3’ R: 5’-TGTAACCATGAGGAACAATACCACC-3’
Negative control siRNA	F: 5′-UUCUCCGAACGUGUCACGUTT-3′ R: 5′-ACGUGACACGUUCGGAGAATT-3′

Note: *NOX4*, NADPH oxidase 4; siRNA, small interfering RNA; F, forward; R, reverse.

### Transfection of plasmids and selection of siRNAs

The CAECs were incubated in DMEM supplemented with 10% FBS, 100 U/mL penicillin and 100 μg/mL streptomycin at 37° C with 5% CO_2_ under a saturated air humidity. The cells were incubated in a six-well plate at a density of 2 × 10^5^ cells/well. The cell confluence reached 90% after 24 h of incubation. Then, 4.0 μg of plasmid and 10 μL of Lipofectamine^TM^ 2000 reagents were added to each well for transfection. The cells were then divided into the siRNA *NOX4*-1 group (transfected with the siRNA *NOX4*-1 plasmid), the siRNA *NOX4*-2 group (transfected with the siRNA *NOX4*-2 plasmid), the siRNA *NOX4*-3 group (transfected with the siRNA *NOX4*-3 plasmid), NC group (exposed to liposomes only) and the blank control group (without treatment of siRNA or liposomes). The serum media were replaced 6 h post-transfection. After cell collection, the total RNA was extracted using a one-step method (Invitrogen, Inc., Carlsbad, CA, USA). *NOX4* expression was determined by RT-qPCR. Ultraviolet (UV) analysis and formaldehyde gel electrophoresis were used to obtain high-quality RNA samples. The PCR primers were designed and synthesized by Invitrogen, Inc. (Carlsbad, CA, USA) (Table 6).

**Table 6:** The primer sequences for RT-qPCR.

**Gene**	**Primer sequence**
miR-363-3p	F: 5'-AATTGCACGGTATCCATCTGTA-3'
	R: 5'-TACAGATGGATACCGTGCAATT-3'
miR-363-3p mimic	F: 5'-AATTGCACGGTATCCA-3'
	R: 5'-GTGCAGGGTCCGAGGT-3'
miR-363-3p inhibitor	F: 5'-UACAGAUGGAUACCGUGCAAUU-3'
β-actin	F: 5’-GTCAGGTCATCACTATCGGCAAT-3'
	R: 5'-AGAGGTCTTTACGGATGTCAACGT-3'

Note: miR-363-3p, microRNA-363-3p; F, forward; R, reverse; RT-qPCR, reverse transcription quantitative polymerase chain reaction.

### Cell transfection and grouping

A CAEC model of CHD (which served as the HCY group) was established after incubation of the CAECs from normal mice for 24 h in 0.25 mmol/L HCY. The untreated CAECs were used as the control group. The DNA oligonucleotide sequence of the overexpressed RNA of the miR-363-3p was synthesized based on a target sequence that augmented the expression of miR-363-3p, from which a double-stranded DNA was generated. The double-stranded DNA was then ligated into the pGCSIL-GFP vector following digestion with the Age I and EcoRI restriction enzymes, after which the positive clones were selected and sequenced. Afterwards, the miR-363-3p mimic, the miR-363-3p inhibitor and a siRNA against *NOX4* (siNOX4) were synthesized, and the liposomes were used to package the miR-363-3p mimic, miR-363-3p inhibitor and siNOX4 (Guangzhou RiboBio Co., Ltd., Guangzhou, China). After the CAECs at passage 3 reached 80% confluence, the diluted liposomes were added to the culture medium, followed by another regimen of 24-h incubation. The CAECs were divided into five groups: the blank group (CAECs without transfection), the NC group (CAECs transfected with the NC sequences), the miR-363-3p mimic group (CAECs transfected with the miR-363-3p mimic), the miR-363-3p inhibitor group (CAECs transfected with the miR-363-3p inhibitor) and the miR-363-3p inhibitor + siNOX4 group (CAECs transfected with the miR-363-3p inhibitor and siNOX4). After transfection, the CAECs were incubated with 0.25 mmol/l HCY for 24 h, after which the subsequent experiments were then carried out.

### Dual-luciferase reporter gene assay

The binding of miR-363-3p to *NOX4* was predicted based on a binding site in the *NOX4* 3’UTR identified using a bioinformatics website (http://www.targetscan.org). The *NOX4* 3’UTR, containing the binding site of miR-363-3p, was then synthesized. Next, a wild type *NOX4* 3’UTR (*NOX4* 3’UTR-WT) was constructed with the pmir-Glo plasmid (Supplementary Table 1). The primer sequence of the *NOX4* 3’UTR-WT was 5'-TAAAGAGGAACAAGTGCAATTTCTAAGACTTAGAAACTCAGCTGAATCAAACAGC-3', or 5'-ATGTGCAATGCTCATGTAGACTGAACTGTGGAGACATAAACTATGACATCATAGCAATGCTGTCTAGATTACAAAGAGTTTTCACAAAACACATACTACAAAAATAAAAATAAAAATAAAC CTCATAAAAAAAAACTTGGGCTTGTCCAGGCTTGATGTGCAATAC-3'. A mutant *NOX4* 3’UTR (*NOX4* 3’UTR-MUT) plasmid was also constructed with site-directed mutagenesis. Detection was performed in strict accordance with the manufacturer’s protocol provided in the plasmid extraction kit (Promega Corp., Madison, WI, USA). Afterwards, the CAECs in the logarithmic growth phase and the HEK-293 cells were seeded into a 96-well plate. Upon attaining 70% cell confluence, the cells were transfected using the Lipofectamine^TM^ 2000 reagent as outlined above. The *NOX4* 3’UTR-WT and miR-363-3p mimic plasmids were then cotransfected into the CAECs and the HEK-293 cells. *NOX4*-3’ UTR-WT + NC, *NOX4*-3’UTR-MUT + NC and *NOX4*-3’UTR-MUT + miR-363-3p mimic groups were prepared as controls and transfected into the CAECs and the HEK-293 cells, respectively. After 6 h of culture in an incubator, the transfected cells were cultured in medium containing 10% FBS for 48 h. A dual-luciferase reporter assay was performed to determine luciferase activity.

### RT-qPCR

Total RNA extraction was performed using a one-step method by TRIzol reagent (Invitrogen, Inc., Carlsbad, CA, USA). The extracted total RNA was analyzed and verified using UV analysis and formaldehyde gel electrophoresis. Then, l μg of total RNA was reversely transcribed into complementary DNA (cDNA). The PCR primer sequences were designed and synthesized by Invitrogen, Inc. (Carlsbad, CA, USA) (Table 2). β-actin was used as an internal reference. The PCR amplification conditions were as follows: predenaturation at 94° C for 5 min, followed by 40 cycles of denaturation at 94° C for 40 s, annealing at 60° C for 40 s and extension at 72° C for 1 min, followed by a final extension at 72° C for 10 min. The PCR products were subjected to agarose gel electrophoresis, and the results were analyzed using the Opticon Monitor 3 software (Bio-Rad Laboratories, Inc., Hercules, CA, USA). The data were analyzed by the 2^-ΔΔCt^ method, as described by the following equation: ΔΔCt = [Ct (target gene) - Ct (reference gene)] _(the experimental group)_ / [Ct (target gene) - Ct (reference gene)] _(the control group)_. This experiment was repeated three times to obtain the mean value.

### Western blot analysis

Total protein was extracted from tissues or cells with radio-immunoprecipitation assay (RIPA) lysis buffer containing phenylmethylsulfonyl fluoride (PMSF) (P0013C, Beyotime Institute of Biotechnology, Shanghai, China). The samples were then incubated on ice for 30 min and centrifuged at 8000 g for 10 min at 4° C to collect the supernatant. The protein concentrations were measured using a bicinchoninic acid (BCA) kit (Wuhan Boster Biological Technology Co., Ltd., Wuhan, Hubei, China). After the addition of loading buffer (30 μg/well), the proteins were boiled at 95° C for 10 min and then separated by 10% polyacrylamide gel electrophoresis (Wuhan Boster Biological Technology Co., Ltd., Wuhan, Hubei, China). Next, the proteins were transferred onto a polyvinylidene difluoride (PVDF) membrane. Subsequently, the membrane was blocked using 5% BSA at room temperature for 1 h and then incubated at 4° C overnight with the primary rabbit anti-mouse antibodies to *NOX4* (1:1000, ab195524), p38 MAPK (1:1500, ab170099) p-p38 MAPK (1:800, ab47363) and β-actin (1:2000, ab8227). The aforementioned antibodies were purchased from Abcam, Cambridge, UK. The following day, the membrane was rinsed three times with Tris-buffered saline-Tween 20 (TBST) (5 min per rinse) and incubated with the goat anti-rabbit secondary antibody at room temperature for 1 h. A chemiluminescence reagent was added to develop the membrane, with β-actin used as an internal reference. The bands were visualized using a Bio-Rad Gel Doc EZ imager (Bio-Rad, CA, USA), and the ImageJ software was used to analyze the grayscale value of the target bands. The experiment was repeated three times to obtain the mean value.

### MTT assay

The CAECs at passage two or three were dispersed into a cell suspension and seeded in a 96-well plate at a density of 5 × 10^4^ cells/well (6 parallel wells for each group). Upon attaining 80% cell confluence, the transfected cells were collected and incubated in 20 μL of MTT solution (Sigma-Aldrich Chemical Company, St Louis, MO, USA) at 37° C for 4 h. After removal of the MTT solution, the cells in each well were treated with 150 μL of dimethyl sulfoxide (Sigma-Aldrich Chemical Company, St Louis, MO, USA) in a shaker for 10 min. The OD of each well was measured at an excitation wavelength of 490 nm using a microplate reader. The average OD values were determined from the mean of three independent measurements. The cell viability was calculated according to the following equation: (OD _the experimental group_ - OD _the control group_)/OD _the control group_.

### H_2_O_2_ and CAT detection

Titanium sulfate colorimetry was used to detect the level of H_2_O_2_ in cells. Fe^2+^ was oxidized by H_2_O_2_ to produce Fe^3+^, and a purple product was formed with addition of xylenol orange in a specific solution to colorimetrically determine the concentration of H_2_O_2_ [52]. The cells were collected into centrifuge tubes, and the supernatant was then removed. A total of 1 × 10^6^ cells were fully lysed using 100-200 μL lysis solution and then centrifuged at 12000 ×g for 3-5 min at 4° C, after which the H_2_O_2_ reagent was liquefied on ice or in an ice-bath. Cell culture medium was added to dilute the standard samples to variable concentrations of 1, 2, 5, 10, 20, 50, or 100 μM. The OD value at the excitation wavelength of 560 nm was measured, according to which a standard curve was plotted. Afterwards, 50 μL of standard sample was mixed with 100 μL of H_2_O_2_ reagent in each test well or tube, which was thoroughly mixed and then incubated for 30 min at room temperature (15-30° C). The level of H_2_O_2_ in the cells was eventually calculated relative to the standard curve.

When H_2_O_2_ is sufficient, CAT can catalyze H_2_O_2_ to generate H_2_O_2_ and O_2_, while the remaining H_2_O_2_ generates a red substance under the peroxidase catalysis [53]. A CAT detection kit (Nanjing Jiancheng Bioengineering Institute, Nanjing, Jiangsu, China) was used to measure the CAT activity in the cells according to the manufacturer’s instructions of the CAT detection kit. The OD value at the excitation wavelength of 240 nm was measured using an ultraviolet spectrophotometer, and the CAT activity was calculated relative to a standard curve.

### Hoechst 33258 staining

Cells in the logarithmic growth phase were isolated using 0.25% trypsin containing 0.02% EDTA. After centrifugation, the cells were resuspended in complete culture medium, followed by transfer of 500 μL of cell suspension into a 24-well plate at a density of 6 × 10^5^ cells/well. The plate was incubated at 37° C with 5% CO_2_. The cells were fixed for 15 min using 4% paraformaldehyde. Subsequently, the cells were stained with Hoechst 33258 at 25° C for five min and dried conventionally. The cells were then observed in five randomly selected fields under an inverted fluorescence microscope. Reduced, dense and brightened cell nuclei were considered apoptotic cell nuclei. The number of positive cells was counted, and the apoptosis rate was calculated as the number of positive cells/total number of cells. The experiment was repeated three times independently to obtain the mean value.

### Tube formation assay

Endothelial cells can form tubular structures. To observe this phenomenon, 5 mL Matrigel (#356230, BD Biosciences, San Jose, CA, USA), which was conventionally stored at -20° C, was thawed at 4° C overnight prior to experimentation. A micropipette (2 mL) and a 24-well plate were also precooled on ice. Then, 500 μL of Matrigel was added to each well and evenly distributed in the 24-well plate. The formation of air bubbles was prevented during this process. Matrigel was then solidified by incubation for 30 min in a 37° C incubator. After detachment, 500 μL of cell suspension (2 × 10^5^ cells/mL) from each group was added to each well. The inoculation was repeated several times for each group. The plate was then incubated at 37° C, and the formation of tubes was observed under a microscope after 15 h of incubation. The number of vascular structures was calculated in three randomly selected fields of view in each well. The experiment was repeated three times independently to calculate the mean value.

### ELISA

Cells in the logarithmic growth phase were detached using 0.25% trypsin-0.02% EDTA. After centrifugation, the cells were resuspended in complete culture medium and then subjected to incubation in a 24-well plate at a density of 6 × 10^5^ cells/well at 37° C in a 5% CO_2_ atmosphere. After the cells adhered to the well, the cell supernatant was collected and stored at -20° C. The levels of ICAM-1 (ab2213, Abcam, Cambridge, UK), IL-6 (ab6672, Abcam, Cambridge, UK), IL-10 (ab100549, Abcam, Cambridge, UK), and IL-1β (ab214025, Abcam, Cambridge, UK) in the cell supernatant were detected in strict accordance with the provided instructions of the ELISA kit. The experiment was repeated three times to calculate the mean values.

### Flow cytometry

After transfection for 48 h, the cells were treated with 0.25% EDTA-free trypsin (YB15050057, Yubo Biotechnology, Shanghai, China). The cells were then collected in a flow tube and centrifuged, after which the supernatant was discarded. According to the protocols for the Annexin-V-fluorescein isothiocyanate (FITC) Apoptosis Detection Kit (K201-100, Biovision, USA), the annexin-V-FITC, propidium iodide (PI), and 4-(2-hydroxyethyl)-1-piperazineëthanesulfonic acid (HEPES) buffer were formulated into the annexin-V-FITC/PI staining solution at a volume ratio of 1:2:50. Next, the cells were resuspended at a density of 1 × 10^6^ cells per 100 μL of the staining solution. After incubation for 15 min at room temperature, the cells were treated with 1 mL of HEPES buffer (PB180325, Procell, Wuhan, China). With an excitation wavelength at 488 nm, a flow cytometer was employed to determine FITC fluorescence by a 525 nm bandpass filter and PI fluorescence by a 620 nm bandpass filter in order to detect cell apoptosis. The experiment was repeated three times [54].

### Statistical analysis

Statistical analyses were performed using the SPSS 21.0 statistical software (IBM Corp., Armonk, NY, USA). Measurement data were presented as the mean ± standard deviation. The data following normal distribution between two groups were compared by an unpaired t-test, and a paired t-test was used for pairwise comparisons. One-way analysis of variance (ANOVA) was utilized for comparisons among multiple groups, followed by Tukey’s test with corrections for multiple comparisons. A value of p < 0.05 was considered to be statistically significant.

## Supplementary Material

Supplementary Table 1
