# Correction for: microRNA-363-3p reduces endothelial cell inflammatory responses in coronary heart disease via inactivation of the *NOX4-*dependent p38 MAPK axis

**DOI:** 10.18632/aging.203865

**Published:** 2022-02-15

**Authors:** Tao Zhou, Suining Li, Liehong Yang, Daokang Xiang

**Affiliations:** 1 Department of Cardiac Surgery, Guizhou Provincial People’s Hospital, Guiyang, 550002, P.R. China

Original article: Aging. 2021; 13:11061–11082.  https://doi.org/10.18632/aging.202721

**This article has been corrected:** A small overlap was found between the CD34-negative and VIII-positive immunochemistry images in **Figure 3**. This occurred because a CD34-negative image was mistakenly placed instead of a VIII-positive image. The authors corrected **Figure 3AB** by using representative images from the original sets of experiments. These corrections do not affect the results or conclusions of this work. The authors would like to apologize for any inconvenience related to this mistake.

New **Figure 3** is presented below.

**Figure 3:**
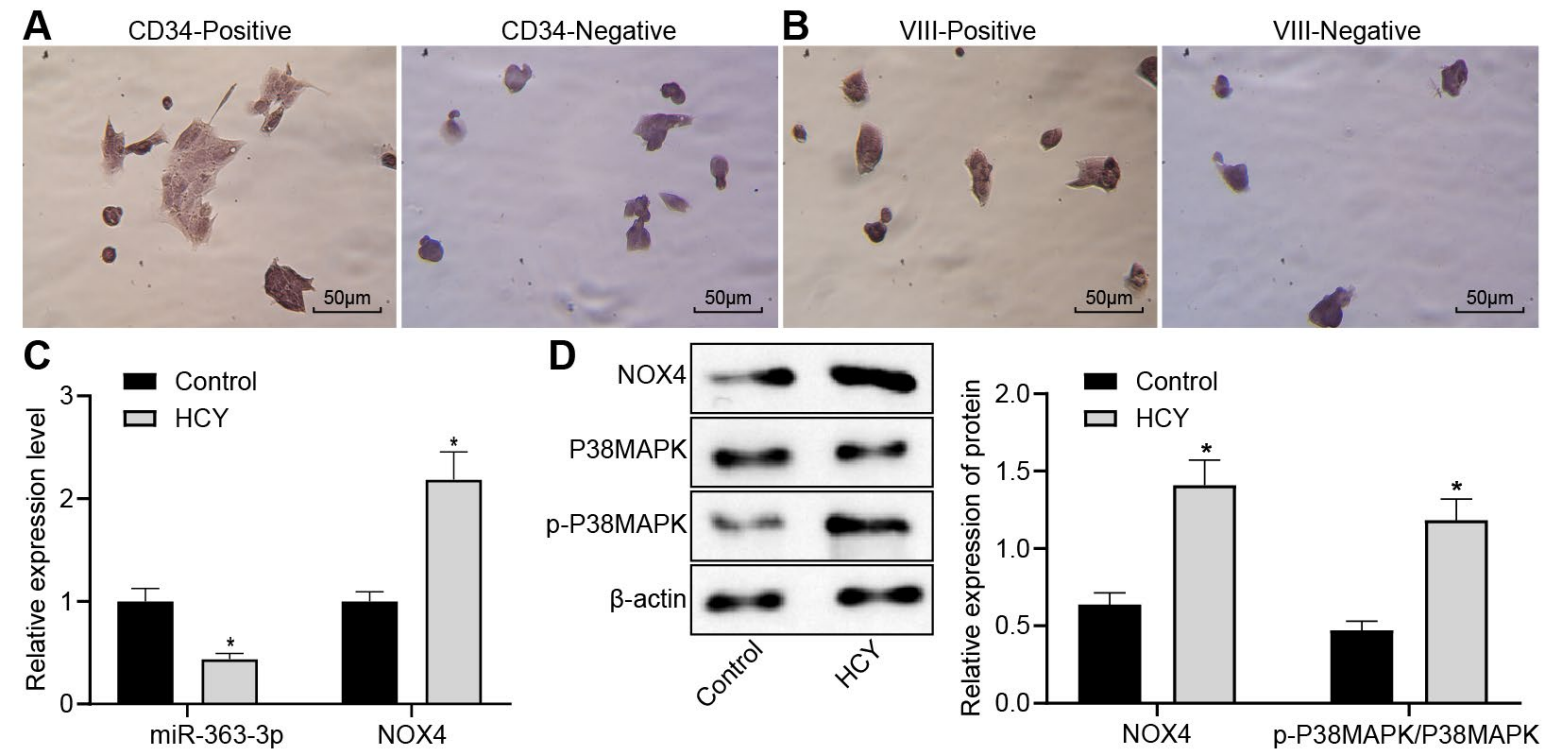
**Reduced miR-363-3p expression and abundant *NOX4* and p38 MAPK expressions in HCY-induced CAECs.** (**A**) positive and negative staining for goat antibody to CD34 (scale bar = 25 μm); (**B**) positive and negative staining for rabbit antibody to Factor VIII (scale bar = 25 μm); (**C**) miR-363-3p expression and *NOX4* mRNA expression were determined by RT-qPCR in HCY-induced CAECs; (**D**) Representative Western blots of *NOX4*, p38 MAPK and p-p38 MAPK proteins and their quantitation in HCY-induced CAECs, normalized by β-actin. The data were analyzed by paired t-test. n = 3. * p < 0.05 vs. the control group; CAECs, coronary arterial endothelial cells; SP, streptavidin-peroxidase.

