# Supplementary material for: microRNA-363-3p reduces endothelial cell inflammatory responses in coronary heart disease via inactivation of the NOX4-dependent p38 MAPK axis
Source: Aging (Albany NY). 2021 Mar 19;13(8):11061–82. doi: 10.18632/aging.202721 (PMC8109087; doi:10.18632/aging.202721)
Supplement: Supplementary Table 1 [file aging-13-202721-s001.pdf]

SUPPLEMENTARY TABLE

Supplementary Table 1. pmirGLO vector multiple cloning site.

**SV40 late poly(A) signal**	
SV40 early enhancer/promotor	106–327
hRluc-neo fusion protein coding region	426–844
Synthetic polyadenylation signal	889–2664
β-lactamase (Ampr) coding region	2728–2776
ColE1-derived plasmid origin of replication	3037–3897
Human phosphoglycerate kinase	4052–4088
promoter	5094–5609
luc2 reporter gene	5645–7297
Multiple cloning site (MCS, Figure 1)	7306–7350

Information of pmirGLO Vector was achieved from https://www.promega.com.cn/Products/Reporter-Assays-and-Transfection/Reporter-Vectors-and-Cell-Lines/pmirGLO-Dual-Luciferase-miRNA-Target-Expression-Vector/?catNum=E1330
